# Business intelligence systems for population health management: a scoping review

**DOI:** 10.1093/jamiaopen/ooae122

**Published:** 2024-11-27

**Authors:** Els Roorda, Marc Bruijnzeels, Jeroen Struijs, Marco Spruit

**Affiliations:** Department of Public Health and Primary Care (PHEG), Leiden University Medical Center (LUMC), The Hague, 2511 DP, The Netherlands; Department of Public Health and Primary Care (PHEG), Leiden University Medical Center (LUMC), The Hague, 2511 DP, The Netherlands; Department of Public Health and Primary Care (PHEG), Leiden University Medical Center (LUMC), The Hague, 2511 DP, The Netherlands; Department of Quality of Care and Health Economics, Centre for Nutrition, Prevention and Health Services, National Institute for Public Health and the Environment (RIVM), Bilthoven, 3721 MA, The Netherlands; Department of Public Health and Primary Care (PHEG), Leiden University Medical Center (LUMC), The Hague, 2511 DP, The Netherlands; Leiden Institute of Advanced Computer Science (LIACS), Leiden University, Leiden, 2333 CC, The Netherlands

**Keywords:** population health management, management information systems, artificial intelligence, business intelligence, data-driven

## Abstract

**Objective:**

Population health management (PHM) is a promising data-driven approach to address the challenges faced by health care systems worldwide. Although Business Intelligence (BI) systems are known to be relevant for a data-driven approach, the usage for PHM is limited in its elaboration. To explore available scientific publications, a systematic review guided by PRISMA was conducted of mature BI initiatives to investigate their decision contexts and BI capabilities.

**Materials and Methods:**

PubMed, Embase, and Web of Science were searched for articles published from January 2012 through November 2023. Articles were included if they described a (potential) BI system for PHM goals. Additional relevant publications were identified through snowballing. Technological Readiness Levels were evaluated to select mature initiatives from the 29 initiatives found. From the 11 most mature systems the decision context (eg, patient identification, risk stratification) and BI capabilities (eg, data warehouse, linked biobank) were extracted.

**Results:**

The initiatives found are highly fragmented in decision context and BI capabilities. Varied terminology is used and much information is missing. Impact on population’s health is currently limited for most initiatives. Care Link, CommunityRx, and Gesundes Kinzigtal currently stand out in aligning BI capabilities with their decision contexts.

**Discussion and Conclusion:**

PHM is a data-driven approach that requires a coherent data strategy and understanding of decision contexts and user needs. Effective BI capabilities depend on this understanding. Designing public-private partnerships to protect intellectual property while enabling rapid knowledge development is crucial. Development of a framework is proposed for systematic knowledge building.

## Introduction

Health care systems are under pressure, due to an aging population, changing health care demands and an increase in technological possibilities.^1^ Simultaneously, in multiple countries, aging causes a shrinking labor force and a growing care demand, rising health care expenditure per capita and causing staff shortage. Furthermore, fragmented governance, resulting in or caused by financing silos, hinders effective response to this development.^2^

Population health management (PHM) is recognized as an approach to simultaneously improve population health, individual patient experience while reducing cost growth, the so called Triple Aim.^3^ Whereas in public health community-wide efforts, policies and programs are used, PHM targets interventions based on specific population needs. PHM is data-driven using data analytics for population needs assessment, population segmentation, risk stratification, impactibility modeling, and evaluation. Successful PHM implementation requires coordinated efforts and data-driven strategies across organizations and sectors.^4–9^ The ultimate goal is to create a learning health system that provides insights into past and anticipated events, helping policymakers to create the necessary conditions to improve population health and enable professionals to take the right actions at the right moment.^10^^,^^11^

In order to realize this data-driven approach, information systems are needed. The PHM papers cited in the paragraph above employ various terms to describe the data-driven approach. Given the different descriptions and definitions, it is assumed that the statement “PHM requires a data-driven approach” is ambiguous.^12^^,^^13^ Laudon and Laudon describe 2 main types of information systems: Transaction Processing Systems that keep track of elementary activities to get patients smoothly through the system, like Electronic Health Records (EHRs) or Care Management Systems, and Business Intelligence (BI) systems which support decision making on different levels.^14^ For PHM, various types of tools may be relevant. Care management systems track patients across different care providers, while risk stratification tools identify risks for populations and individuals and biobanks facilitate research into genetic factors. BI systems provide automated and ongoing insights from collected data to policymakers and professionals, focusing on aspects such as the health of diverse populations and the outcomes of interventions. This can lead to overlapping functionalities between different types of systems.

The definition of BI develops since the first use in the 1950s.^13^ According to Brooks et al, the current definition contains organizational processes, business processes and data governance, next to the technical tools and processes to store and use data from multiple sources in a data warehouse with graphical tools.^15^ Volumes of data processed by BI systems grow with the introduction of eHealth devices and the use of unstructured data increases. These developments require new approaches, both in technical and in organizational terms. Another crucial factor for a successful BI implementation is the understanding of and the alignment with the decision context.^16^ Decision context refers to the circumstances, setting, or environment within which a decision is made. This context could include relevant information, constraints, goals, risks, stakeholders, and other elements affecting the decision-making process. The value of BI capabilities depends on their usability in this process.

Supporting decision processes with BI and creating the needed BI capabilities, in technical and organizational terms, is complex within a health care organization.^17^ To realize a data-driven approach within a PHM context is even more complex. In many countries the required source data from EHRs are organization specific, resulting in fragmented insights into individuals’ health. The governance needed to develop, implement, and support the BI system interorganizationally is therefore complex. Furthermore, multiple interacting barriers ranging from technical to intangible issues,^18^ such as legal, ethical, and political barriers, impede progress in population health data sharing.

Often, healthcare BI systems are developed commercially or by health authorities, potentially limiting opportunities for international knowledge building. Intellectual property of commercial products may lose value when information is shared, while authorities have different priorities. Not publishing about these tools leads to intransparency, limited validation, and the risk of many premature and overlapping tools. Given the rapid pace at which most countries are confronting a healthcare crisis and the complex context in which a BI system for PHM must be implemented, sharing evidence is essential. Despite organizational differences between countries, overarching lessons still can be learned.

In this paper a first exploration is provided to investigate which scientific knowledge is available regarding BI systems for PHM. A better understanding of the decision context and capabilities of BI in the PHM setting is needed. Therefore this review creates an overview of existing mature systems described in scientific literature, as such an overview of BI systems for PHM is currently missing.

The following research questions are addressed:

1. In which decision contexts are scientifically described more mature BI systems for PHM used?
2. Which BI capabilities, in terms of techniques and organization, are realized to develop and operate these BI systems?

## Methods

### Selecting mature BI systems

#### Search strategy and information sources

To identify as many initiatives as possible that implemented a BI system for PHM, the scientific literature was reviewed. The PRISMA framework guided the process to ensure validity and reliability.^19^ A librarian-assisted search strategy was developed for PubMed, Embase, and Web of Science for articles published from January 2012 through 18th of November 2023. Diverse search terms from both technical and application perspectives mitigated terminological variation risk. The search string combined technical keywords, including “information system,” “business intelligence,” and “learning health system,” with application-oriented keywords such as “population health” and “accountable care organizations.” Clinical studies were excluded. The full search strategy is reported in the Supplementary Appendix S1.

Additional relevant articles were identified through backward and forward snowballing of relevant articles and systematic reviews. Also initiatives from the information and research chapters of the SELFIE2020 thick descriptions were used as search term to find publications about the initiatives.^20–27^

#### Study selection

First, duplicates were removed. Articles were excluded if they were commentaries, reviews. Two researchers independently screened the remaining articles for eligibility based on the title and abstract. Eligible articles, written in English, German, or Spanish, met the following criteria:

1. Describe an automated information system or scalable idea/prototype, not a one-time analysis or model development/validation;
2. Specify that the information system is designed to achieve one or more of the PHM-goals;
3. Describe the (intentional) use of the information system in an integrated care setting;
4. The (intended) system is a BI system, not an EHR, Personal Health Record or a survey system.

Articles were grouped in the categories eligible and non-eligible. Differences were discussed and, if there remained any doubt, the full-texts were retrieved to reach consensus on whether or not to include the article.

#### System maturity

Comparing existing information systems requires understanding of the maturity of the systems, since a system in production has other characteristics then a system in development. The Technology Readiness Levels (TRLs) methodology from the work of John C. Mankins^28^ was translated to BI systems for PHM in Table 1. For this review the more mature systems, TRL 6 or higher, were included with the completeness of information about the systems in mind.

**Table 1.** Translation of technology readiness levels to the PHM domain.

Level	Phase	General description	Translated description for PHM information systems^b^
TRL 1^a^	Discovery	Basic principles observed and reported	Useful techniques/algorithms are described, eg, distributed computing as a concept.
TRL 2		Technology concept and/or application formulated	Potential application in PHM for a technique or algorithm is described
TRL 3		Analytical and experimental critical function and/or characteristic proof-of-concept	A proof of concept of a technique or algorithm is made in a research context
TRL 4	Development	Component validation in a laboratory environment	A development version of a (sub)system is made in a research context with real data collected in a project setting
TRL 5		Component validation in relevant environment	A development version of a (sub)system is tested in a research context with real data in a scalable setting, data collection largely automated
TRL 6		System/sub-system model or prototype demonstration in a relevant environment	A development version of a (sub)system is tested in a region with real data which are refreshed frequently for a test population and is tested by a number of professionals and citizens
TRL 7	Demonstration	System prototype demonstration in the expected operational environment	The system is tested in a region with real data of the subpopulations and is tested by a number of organizations, professionals, or citizens in the desired workflow
TRL 8		Actual system completed and “qualified” through test and demonstration	The improved system is used in a region with real data of the whole population and is used by most organizations, professionals, and patients
TRL 9	Deployment	Actual system “flight proven” through successful mission operations	Through use of the system, health is promoted and inequalities across whole populations are reduced

a TRL: Technology readiness level

b PHM: Population Health Management

### Decision context: goals and user groups

To analyze the decision context of the BI system requires understanding of the goals for which the system is used as well as insight in the type of person who makes the decision. Unless there is ambiguity with the definition of PHM,^3^ there is consensus on several overarching elements, such as impacting social determinants and focusing on population differences. The following PHM objectives, combined from the work of Washington and Steenkamer,^29^ were used to group the goals of the BI systems:

- *Patient Population Identification* is the capability to find and define specific populations for a specific PHM initiative.
- *Triple Aim Assessment* represents the determination of the health for a specific population from different angles (eg, self-reported health, claims, clinical documentation) and the assessment of the experience of care (quality and satisfaction) and the costs of care.
- *Risk Stratification* is the process of dividing populations into meaningful risk categories for person-centered intervention targeting.
- *Engagement* refers to the active involvement of individuals in their health care decisions and management.
- *Patient-centered Interventions* are defined for the different subpopulations in the whole care continuum, from prevention to palliative care, based on the expected impact.

*Impact Evaluation and quality improvement* is the ability to evaluate the outcomes of interventions to be able to define improvements.

Multiple user groups may benefit from the BI systems to reach the PHM goals listed above. The users were divided in 4 groups, based on the levels of the Rainbow model^30^:

- *Clinical*: Health care professionals use information for the coordination of care for an individual patient.
- *Professional*: Health care professionals exchange information with other professionals within and outside their organizations to reach the Triple Aim for specific groups of patients.
- *Organizational*: Health care organizations use information to collaborate to improve health on the organizational level to reach the Triple Aim.
- *System*: Information is used to design the health/social system, for example, via paying models and laws and regulations.

### BI capabilities

The systems and their governance are evaluated on the following categories and subcategories^15^^,^^31^:

- *Technical solution* consists of the following 4 components: General Architecture and Infrastructure, Data Modelling, Data processing, and BI Applications.
- *BI Organization and Processes* dimension comprises the subcategories: Development and Operation.

Within these subcategories more detailed aspects were defined: General Architecture and Infrastructure was split into the type of BI system, the kind of source data used and the physical security measures. Data modelling is divided into the design levels used, the database (or other) technology used to model data and the data standards (eg, ICD-10) used. Data processing contains the load frequency and the amount of data loaded in terms of population size. BI applications describes whether retrospective, real-time of prospective data is used, which algorithms are used and which front end tool is provided to the end users. Development refers to insights about project management, the development team, the procedural approach adopted and the funding for the development phase. Operation contains the number of users, the business processes in which the system is used, the measures and processes to guarantee privacy, the service processes and the long term funding.

### Data extraction and synthesis

The following information was extracted for all selected papers: first author, year of publication, country, publication type, name of initiative, and description of maturity. Next, the TRL of initiatives was determined and reviewed. The full-text articles of initiatives with TRL 6 or higher were assessed by 2 reviewers on descriptions of the decision context and the BI capabilities.

## Results

### Selection of mature BI systems

**Figure 1.**
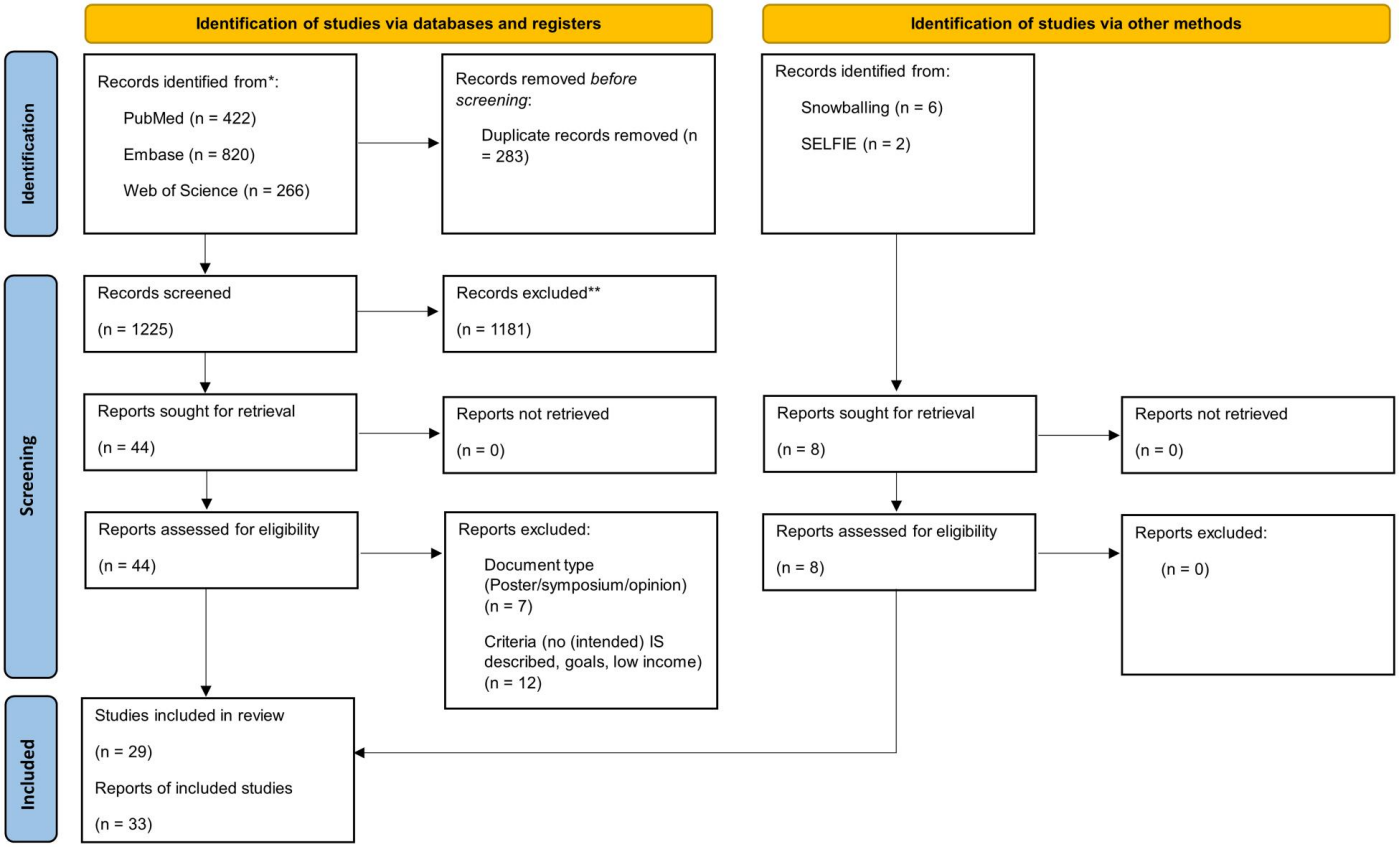
PRISMA flow chart.

Figure 1 shows the study selection flow diagram. 1225 articles were identified from which 44 publications were assessed for eligibility. Forward and backward snowballing yielded an additional 8 papers. After removal of duplicates and non-eligible articles, the final sample consisted of 33 studies containing 31 articles and 2 book chapters. The studies described 29 unique initiatives.

Table 2 contains the 29 unique initiatives and their TRL. The initiated framework for maturity was quite applicable, whereas the systems are relatively well-described. However the roadmap for the intended implementation, the desired usage in practice and the value for the health of the population is often lacking, which makes it hard to classify the exact maturity level. The decision tree used by the reviewers is included in the Supplementary Appendix S1. Eleven systems were classified with TRL 6 or higher: three systems were categorized as having demonstrated value (TRL 9), five systems were in the demonstration phase (TRL 8), and three systems were at the end of the development phase (TRL 6). The remaining 16 systems are classified in lower TRL levels. Notably, 2 of the 3 TRL 9 initiatives show a symbiotic relationship between transactional and BI characteristics, as the insights generated by BI have been successfully integrated into the operational workflows.

**Table 2.** Information systems and their TRLs.

Initiative name, Year, Country	Technology readiness level (TRL) estimation arguments	TRL
Care Link, 2017, United States^32^	*Care Link interventions have shortened hospital lengths of stay and directed a greater volume of patients to return home directly after their hospital stays *There was a reduction in readmissions after 90 days	9
CommunityRx, 2016, United States^33^^,^^34^	*Implementation at 33 clinical sites for 37 prevalent health and wellness conditions *253 479 personalized HealtheRX “prescriptions” were generated *Participants found HealtheRx very useful in a 20 month evaluation period around 2013 *CommunityRx has been developed and iterated over more than a decade using an asset-based community-engaged approach	9
Gesundes Kinzigtal, 2016, Germany^35^^,^^36^	*The very high levels of investment have paid off and without this investment the ever growing 7 figure savings contract results produced by GK would not have happened^a^	9
Andalusian health population database, 2020, Spain^37^	*The system has more than 300 users with a management and public health profile *Started giving access to primary care professionals with access to their patients’ nominal data to support care management *Reached value for population health not described	8
GARDE, 2022, United States^38^	*Multi-site validation demonstrated GARDE’s standards based design identifying 42 000 patients who met criteria for genetic testing *Reached value for population health not described^b^	8
Rhône-Alpes regional health platform, 2012, France^39^^,^^40^	*111 sites were feeding regional records in 2011 *Reached value for population health not described	8
The Hub Population Health System, 2012, United States^41^	*Coverage in 2012 will be 2.5 million New Yorkers *Reached value for population health not described	8
PHATE, 2020, United States^42^	*Pricing information is available on the website *PHATE would benefit from further evaluation of its utility for education and clinical care *Reached value for population health not described^c^	8
Estonian biobank, 2015, Estonia^43^^,^^44^	*Future plans include linking the genome center database with the national health information system in real time, as well as using the data form piloting personalized medicine *Estonia had just finished 2 pilots on personalized medicine (manuscripts in preparation) *In the longer term the plan is to genotype most of the population allowing the development of more precise genetic instruments for medical decision making	6
PopHR, 2017, Canada^45^	*The system is available for a small number of test users	6
Primary Sense, 2020, Australia^46^^,^^47^	*Formal live testing of the system was completed in 9 practices *The next phase of this project is now underway to further trial the scalability and change management requirements for full implementation	6
National RHS database, 2016, Singapore^48^	*Data linked in a anonymized database *Used by researchers	5
Query Health, 2014, United States^49^	*Deployed at 3 pilot sites (1) New York 3 test systems (2)Within the MDPHnet project, Query Health is still used (3) In FDA’s mini sentinel project 3 months successful test run *The present reference implementation provides a common set of components^d^	5
SeVa, 2021, United States^50^	*The digital platform developed is currently being evaluated within a hospital setting *Does not involve human subject use and evaluation at this time	5
MEMPHI-SYS, 2023, United States^51^	*Data warehouse created for MEMPHI-SYS will promote broader application of practical, hypothesis-driven interventions to reduce health and social risks in disadvantaged neighborhoods *Collaboration with another institution holds promise to reduce longstanding health disparities well past the current pandemic’s end	5
Mental Health, 2023, Italy^52^	*Four MHDs from three Italian participated in this pilot project to explore geographic variations in care and the feasibility of introducing bundled payments^e^	5
EURO-HEALTHY PHI, 2020, Europe^53^	*The EURO-HEALTHY research project collected and systemized a vast amount of data for 80 indicators *Developed an open access user-friendly WebGIS platform *The evidence has the potential to create windows of opportunity—which could evolve into learning networks^f^	4
MDPHnet, 2014, United States^54^	*2 proof-of-concept queries demonstrate MPDHnet’s capacity to enhance routine public health surveillance and evaluation *There are several options for funding a network like MDPHnet	4
POHEM, 2015, Canada^55^	*Most examples discussed in paper come from the POHEM model of cardiovascular disease *Model has been used to research the projected prevalence of risk factors for heart disease	4
Screening and early warning health management system for populations at high risk for depression, 2022, China^56^	*Study and design a screening and early warning health management system	4
Shielding COVID-19, 2020, United Kingdom^57^	*Study on the use of PHM methods on a linked dataset^g^	4
Urban Population Health Observatory (UPHO), 2021, United States^58^	*A prototype design to present features	4
An infrastructure for real-time population health assessment and monitoring, 2012, Canada^59^	*Describe the design and prototype implementation of a novel public health infrastructure	3
Data linkage of primary and secondary data, 2014, Germany^60^	*Conclusions are draw regarding future application areas and options of small area health services research and specific examples are provided	3
Similar patients dashboard infectious diseases, 2016, United States^61^	*Extracted similar complex patient information from a clinical database and presented in a population information display in an exploratory study	3
Toward a fine-scale population health monitoring system, 2021, United States^62^	*Analysis of the intersection of Race/ethnicity (from EHR and self-reported) and genetic ancestry *Apply a framework to detect fine-scale population structure in a Biobank^h^	3
Data and knowledge standards for learning health, 2018, United States^63^	*Identified the data-dependent activities and the type of data standards required	2
Population health monitoring, 2020, Netherlands^64^	*The model provides insight into the various steps and actors involved and into the relationships between population health monitoring and relate concepts such as health information (systems)	2
Advancing the evolution of healthcare, 2014, United States^65^	*Presents an example of information technology integration	1

a GK: Gesundes Kinzigtal

b GARDE: Genetic Cancer Risk Detector

c PATHE: Population Health Assessment Engine

d FDA: Food and Drug Administration

e MHDs: Mental Health Departments

f WebGIS: Web-based Geographical Information System

g PHM: Population Health Management

h EHR: Electronic Health Record

#### Systems with proven value

The 3 systems with TRL 9 differ from each other. The Care Link system supports data-driven care management for many diseases and chronic conditions to ensure that the patient experience is seamless in the transition between inpatient and outpatient settings. The system provides longitudinal care management to address evidence-based secondary prevention goals, real-time admission, discharge and abnormal laboratory test-result notifications, and proactive real-time identification of individuals most at risk or in need of attention.

CommunityRx is an e-prescribing module for community care based on integrated data. Prevalent social and medical conditions in the population are mapped to community resources. Using a smartphone application, youth workforce walked block by block, gathering data of community service providers. Clinicians can prescribe community resources in a patients neighborhood from their electronic health record.

Gesundes Kinzigtal is a region that is using a BI system in an integrated care setting. Data from various data sources are linked in a Data Warehouse, prepared, enriched and used for management support via a BI front-end: starting with the project preparation and development via the ongoing project management up to a final evaluation, eg, by using benchmarks of costs, patients satisfaction, and patients incapability to work between practices.

#### Systems in the demonstration phase

The 5 systems with TRL 7 and 8 are divers as well. The Andalusian health population database is an information system that connects data from multiple health records to improve assistance to patients, health services administration, management, evaluation and inspection, as well as public health and research. The Genetic Cancer Risk Detector (GARDE) platform is a scalable standards-based clinical decision support (CDS) approach to identify patient cohorts for PHM and maximize opportunities for multi-site dissemination. The Rhône-Alpes regional health platform contains 2 information systems. The first system contains distributed medical records which store metadata with connectors to different EHRs. The second system is an epidemiological platform with de-identified data. The Hub Population Health System provides services for distribution of query reports, alerts and secure messages to providers. The Population Health Assessment Engine (PHATE) is a social deprivation index—a small area index associated with poor health outcomes, disease prevalence, and increased costs. Integration of PHATE within an EHR provides a reliable first-pass assessment of patient risks based on where they live.

#### Systems demonstrated in a relevant environment

Three initiatives with TRL 6 are identified. The Population Health Record (PopHR) is a web-based application designed to facilitate the effective use of population health information by public health professionals and to support evidence-based decision-making. Primary Sense focuses on finding patients in general practitioner data which will benefit from an intervention at the clinical level. Another system in development is the Estonian biobank whose database allows a wide spectrum of genomic and epidemiological research to be conducted. Future plans include linking the genome center database with the national health information system in real time, as well as using the genetic data and the technical infrastructure available for piloting personalized medicine.

### Decision context of included systems

As can be seen in Table 3 in the descriptions that are literally taken from the publications, the coding was challenging due to variations in terminology.

**Table 3.** Selected BI systems and their decision context.

Initiative name	Decision context	Goals	User group
		Patient Population Identification Triple Aim Assessment Risk Stratification Engagement Patient-centered Interventions Impact Evaluation and quality	Clinical Professional Organizational System
Care Link	*Care coordination to fill gaps in the health care system and to get people the right care at the right time, in the right place and with the right community resources	N/A N/A Identify people at risk or clinical scenarios that require attention, in combination with a comprehensive care management program, based on clinical information, utilization history and demographic data Insufficient information to judge (Care Link leverages virtual and embedded staff to drive patient engagement) Technology based care management tools could be used to customize the clinical and social interventions Several graphs show impact evaluation, information whether impact evaluation is part of the information system is not available	Care management team receives notifications Care managers provide clinical updates to clinicians Care management team reach out to hospitals or emergency departments N/A
CommunityRx	*e-prescribe community resources for basic, wellness, and disease self-management needs	N/A N/A N/A An IT solution that enabled clinicians to e-prescribe community resources for basic, wellness, and disease self-management needs Create a personalized list of community resources N/A	More than 1600 providers (physicians, nurses, and other staff) were trained. N/A N/A N/A
Gesundes Kinzigtal	*Improving the health of the population *Economic efficiency of healthcare *Improving patients experience	N/A Health care outcomes, divided into the Triple aim dimensions (eg, mortality, years of potential life lost, contribution margin, patient experience) N/A N/A N/A It supports the entire Plan-Do-Study-Act management cycle	General practitioners and specialists gets performance management information N/A Hospitals and pharmacies, etc., get management information; Analysis are used by the project group Network management levels gets performance management information
Andalusian health population database	*Reconstruct health biography *Health planning *Health management *Health research *Strategic planning *Program contracts (meso) *Clinical management (micro)	The listings contain population characteristics and prevalence of chronic diseases The listings contain resource use and costs N/A N/A N/A N/A	Primary care professionals have access to patients’ nominal data to support care management N/A Decision support for program contracts More than 300 users with a management and public health profile; Possibilities for health planning, management, strategic decision making and research
GARDE	*Identify cohorts for PHM	Patient data warehouse to select target populations. Provides information on family history, genetic counseling encounters, and outreach status N/A N/A N/A N/A N/A	Results are written to the EHR PHM system^a^ N/A N/A N/A
Rhône-Alpes regional health platform	*Allow sharing of EHRs with other practitioners to coordinate patient care *Regional epidemiological platform	N/A Metadata is available for collection public health indicators N/A N/A Sharing EHR, coordinate patient care N/A	N/A Allow sharing of EHRs with other practitioners to coordinate patient care N/A Metadata for collection public health indicators
The Hub population health system	*Evaluate population health and quality improvement activities *Targeted alert campaign	Targeted alert campaigns for public health emergencies Examine distribution of disease. Monitor health care outcomes N/A N/A N/A N/A	Alert per patient in EHR N/A N/A Distribution of disease to inform program planning
PHATE	*Adjust payments *Contribute to public health surveillance *Organize care around hot spots *Assess patient risk *Connect with community organizations	Identify patients with social risk factors Measure quality corrected for risk factors, provide public health data Assess risk by geographic distribution A free national directory of community resources is integrated N/A N/A	N/A N/A Organize care around hot spots, assess patient risk, connect with community organizations Adjust payment and quality for social inequalities, contribute to public health surveillance
Estonian biobank	*Calculate disease risk and likely drug response	N/A Cost-effectiveness analysis Develop risk predictors by integrating genetic, health, and environmental factors N/A Medication response prediction N/A	Tools to be used in clinical practice are developed N/A N/A Develop predictors for the risk of a disease; cost-effectiveness analysis
PopHR	*The exploration of available indicators and knowledge *The analysis and visualization of selected indicators for a defined population	N/A Measurement and monitoring of population health and health system performance Identifying factors for health status. The framework consists of a set of ontologies which are used in AI to capture knowledge N/A N/A Evaluate effects of public health interventions and other programs	N/A N/A N/A System is used by public health practitioners
Primary sense	*Reports of patients who are likely to benefit from specific interventions *Medication alerts *Comparison of the population health profile across practices *Using evidence based risk algorithms	Identify patients with risk factors N/A Quantify risk for individual patients N/A N/A N/A	Realtime medication safety alerts N/A Reporting tool for practices N/A

a EHR: Electronic Health Record; PHM: Population Health Management

Looking at the PHM goals, 5 initiatives are able to identify populations, from which 4 are able to find persons with specific characteristics (Primary Sense, PATHE, GARDE, and Andalusian health population database) and 2 initiatives can identify people for alert campaigns (The Hub Population Health System and Primary Sense). Most applications are on the clinical level, some are on the organizational or system level.

Among the evaluated initiatives, Gesundes Kinzigtal offers comprehensive insight into all facets of the Triple Aim model. The Hub Population Health System and PATHE primarily focus on health assessment and quality evaluation. PopHR concentrates on health evaluation coupled with cost analysis, whereas the Rhône-Alpes regional health platform focuses solely on health assessment. The Andalusian health population database assesses cost factors.

Risk stratification is performed on the individual level in Primary Sense and Care Link and on a population level in PopHR, Estonian biobank, and PATHE. The methods used for risk stratification are diverse, in the Estonian biobank DNA is used, PopHR uses ontologies, PATHE uses geographic information, and Care link uses clinical information, care utilization, and demographic data.

The use of systems for engagement is limited. CommunityRx has as main goal to provide self-management needs and this system seems implemented in PATHE as well. The Care Link publication mentions engagement without further information.

Care Link, CommunityRx, and the Estonian biobank use their BI systems to customize interventions to the patients’ needs and characteristics. These initiatives all operate on the clinical level.

A BI system is used in only 2 initiatives for impact evaluation, which is less than anticipated, whereas BI is commonly used in Plan-Do-Check-Act cycles. Gesundes Kinzigtals system is used in this cycle and PopHR evaluates interventions.

The 11 BI systems are used by various groups of users, only PHATE reports explicitly on user group levels. Many initiatives operate on the clinical level with different goals, from care management to generating alerts to prescribing. Two initiatives are meant to inform other professionals: Care Link and the Rhône-Alpes regional health platform. Many systems provide decision support information on the organizational or system level. Only PATHE provides information to adjust payment.

### BI capabilities

Table 4 describes the technical solution and the organization and processes for the mature systems. The technical solutions were found to vary significantly as well. Perhaps the most notable observation is the lack of description regarding the technical considerations. Often, only the final solution is described without providing insights into the design process. The main finding on organization and processes is that this category is described to a very limited extent. Not a single paper provided a comprehensive insight into the development processes and operational processes.

**Table 4.** Selected BI systems and their capabilities.

Initiative name	Technical solution				Organization and processes	
	**General architecture and infrastructure** System type Data used Security	**Data modelling** Design levels Database tool Standards	**Data processing** Frequency Population size	**BI applications** Retrospective/real time/prospective Algorithms Frontend tool	**Development** Management Project team Development processes Funding development	**Operation** Number of users Business processes Privacy Service processes Long term funding
Care Link	BI, EHR^a^ Various sources including clinical information, utilization history, demographic data N/A	Operational data store with business logic N/A N/A	Near real-time 48 000	Retrospective/real time/prospective Predictive signal detection and analysis for future utilization N/A	N/A N/A Started with one disease, scaled up to multiple diseases 10m grant to design a generalizable, scalable, and replicable IT-driven care model	300 physicians Longitudinal care management N/A N/A N/A
CommunityRx	E-prescribing module within EHR with link to communityRX system Community services locations, and surveys, EHR data Secure webcall to CommunityRx server	N/A N/A N/A	N/A 113 000	Retrospective Algorithm that generates a personalized list of community resources near the participant’s home address Implemented in EHR	N/A N/A Rapid cycle iteration methods Health innovation fund	1600 providers, Clinical visits N/A N/A More investments were made
Gesundes Kinzigtal	ICT infrastructure with BI system Claims data from health insurers from al care sectors, survey data External data protection supervisor	Integration in a datawarehouse SQL server^b^ N/A	Monthly update 33 000	Retrospective Risk adjustment Deltamaster BI	N/A N/A Development with end users from the start 9 year contract with investment company	N/A PDSA cycle^c^ Person based pseudonymized N/A N/A
Andalusian health population database	BI system Inpatients, outpatient mayor surgery, hospital emergencies and medical day hospital, mental health, analytical and image tests, vaccines, renal patients and pharmacy Secure authentication from corporate network	Database, application layer, presentation layer Oracle ICD, HCUP, Nanda Nic Noc^d^	N/A 8.5 million	Retrospective Automatic coder for clinical diagnosis Microstrategy BI/web, expert users SQL/R	Managment and steering committee, for governance. Technical monitoring committee and support groups for specific aspects N/A N/A N/A	300 N/A Pseudonymized data N/A N/A
GARDE	Component architecture Secondary care N/A	CDS Hooks server, Population Coordinator, EHR data repository N/A FHIR, CDS hooks^e^	N/A >1 million on 3 sites	Retrospective Familiar Cancer Detection Algorithm Communication request (FHIR)	N/A N/A N/A Grant IT for cancer research	N/A N/A N/A N/A N/A
Rhône-Alpes regional health platform	Web-based shared health record without interfering in current information systems, epidemiologic platform All health services data Authentication with professional electronic card	Meta meta (definition of the abstract structure), meta (abstract structure), business level N/A French DMP, ICD-10^f^	N/A 2.6 million	Retrospective, real time Patient identification over EHRs Webbased	The steering committee monitors the progress of projects. Ideas started from observations in the field by practitioners. Key was involvement of end users. Development done by members with industrialists Tools are developed with public funds	N/A Epidemiological analysis, piggybacked for other purposes like a capacity marketplace Professionals authorized by patients; option to refuse for epidemiological use N/A N/A
The Hub Population Health System	Distributed queries and alerts Ambulatory primary care practices data Aggregated data secure https	EHR clinical data repository, Hub, datawarehouse N/A N/A	Nightly 2.5 million	Retrospective N/A N/A	N/A N/A N/A N/A	N/A Alert campaigns, real time population health monitoring Aggregation before sending to the Hub N/A N/A
Estonian biobank	Biobank linked with other registries Genomics, national health registries National electronic identification card	N/A N/A N/A	N/A 202 000	Retrospective N/A N/A	N/A N/A N/A	N/A N/A Legal framework, informed consent N/A N/A
PHATE	BI tool including social determinants linked with EHR Community Vital Signs, diagnoses, quality measures, addresses N/A	N/A N/A N/A	N/A N/A	Retrospective N/A Geospatial reporting tool	N/A N/A N/A Funded as scholar program	N/A N/A N/A N/A N/A
PopHR	A semantic web framework that makes population health indicators available Health insurance provider data, retail data, surveys N/A	Domain ontologies, application ontology upper ontologies N/A Reusing ontologies	Near real time 1 million	Near real time Description logic reasoners Webbased application	N/A N/A N/A N/A	N/A Monitor public health, evaluate effects of health interventions N/A N/A N/A
Primary Sense	Reports and (real-time) alerts Data from general practitioners N/A	N/A N/A ICPC, ICD	N/A N/A	Retrospective, real time ACG^g^ N/A	N/A A group of local general practitioners and other experts N/A N/A	N/A Prescribing, prevention N/A N/A N/A

a BI: Business Intelligence; EHR: Electronic Health Record

b SQL: Structured Query Language

c PDSA: Plan-Do-Study-Act

d ICD: International Statistical Classification of Diseases and Related Health Problems; HCUP: Healthcare Cost and Utilization Project

e CDS: Clinical Decision Support; FHIR: Fast Health Interoperability Resources

f DMP: Shared Medical Record (in French)

g ACG: Adjusted Clinical Groups

To analyze in more detail, in General Architecture and Infrastructure there are a number of more classical BI systems and a number of distributed systems, the other systems have a customized architecture. Most systems use EHR data from general practitioners or hospitals combined with other sources. The security of the systems is ensured through various technical and process-based measures. Data modelling is described for most of the systems and is diverse as well. The design levels and tools used are unique for each system. Many of the systems use international standards like ICPC or ICD. The loading frequency is often not specified. Population sizes seem either small and focused on specific geographical areas or populations (<100 000) or more broad (>1 million) and are always specified. Most systems contain retrospective data, some systems provide (near) real-time data and one system contains prospective information. Many systems use different types of algorithms. The front-end tools vary significantly and widely used BI and analytics solutions and vendors, such as Microsoft, Tableau/Salesforce, and Qlik, appear to be limited in their adoption.

Organization and processes are unique for each initiative and sparsely described. Themes such as governance, development, funding, and operational processes are not described in most cases. The number of users is reported in some cases. The business processes are almost always described and in line with the previously identified PHM goals. Privacy measures are partially described and implemented in various ways, including aggregation, pseudonymization, and consent.

## Discussion

This was the first study that investigated the available scientific knowledge regarding BI systems for PHM, especially regarding their decision contexts and BI capabilities. PHM has several goals to improve the health of populations: patient population identification, triple aim assessment, risk stratification, engagement, patient-centered interventions and impact evaluation, and quality improvement. The data-driven approaches for these goals were extracted from the initiatives, resulting in diverse goals and user groups. However, efforts are often fragmented, addressing only isolated elements of PHM rather than adopting a comprehensive approach. For example CommunityRx focusses on engagement and tailored interventions on the clinical level whereas Gesundes Kinsigtal focusses on planning and monitoring interventions. Descriptions on organizational aspects of the initiatives were extremely limited, hindering an understanding of the organizational context. In many cases, there is no long-term funding and vision described, raising questions about whether these initiatives can achieve sufficient maturity and impact. Furthermore, limited consideration is given to technical aspects of scalability to other regions and countries, such as using standards.

Despite PHM being called a data-driven approach it is observed that current research on the data dimension of PHM is very limited, lacks coherence and a unified approach. There is a lack of structured methodologies and harmonization among studies. This diversity yields a lack of scientific knowledge about the technical and organizational aspect and decision context of a PHM data infrastructure. Inadequate implementation of PHM ensues, leading to a suboptimal realization of the approach’s full potential.

Private organizations and authorities often prioritize other interests above knowledge sharing. Presently, their publications predominantly focus on isolated components of a product, such as (predictive) models. There is considerable potential to share more about the contextual aspects of the product. Firstly, exchanging insights on applications where BI systems benefit populations would be valuable, given that only 3 such cases have been identified. Secondly, disseminating knowledge on integrating BI systems into work processes and culture is crucial. Thirdly, discussing approaches to development and (data) management processes, and how these might differ when collaborating with a commercial partner or an authority, is important. An Implementation of a Data-Driven Approach (IDDA) framework for reporting the data-driven approach of PHM initiatives should lead to more comprehensive and comparable publications. Part of the framework should be a translation from PHM goals to decision contexts and the needed BI capabilities. The framework could also describe whether one should begin with detailing the desired work process, as seen in the 3 TRL 9 initiatives, or with the technology, which is often observed in the other initiatives. Developing and using a framework could ultimately lead to a learning community that develops more valuable BI systems. Special attention should be given to the unity of language, since terminology usage is currently diverse. While various publications have been observed where BI was not used as a term despite being relevant, and vice versa. The coherence with systems like care coordination, risk stratification tools, and biobanks should be described as well, as BI functionality overlaps with these systems in certain decision contexts as can be seen with Care Link and CommunityRx.

### Strengths and limitations

Limitations of this study include that relevant papers might be missing, for example caused by the use of different terminology. Nevertheless multiple search terms in both the systems and application domains were used. We intentionally focused solely on scientific literature, which biases our view towards larger integrated or academic systems. This underscores the need for new arrangements within public-private collaborations about sharing knowledge more scientifically. Comparing systems across countries with diverse approaches was intentionally pursued to extract overarching findings. Although a systematic approach was attempted, the process is substantially complicated by inconsistent reporting.

One of the major strengths of this research is the systematic approach used to identify and select relevant articles. By using the PRISMA guidelines, it is ensured that the review process was comprehensive, objective, and reproducible. Another strength of this research is a multidisciplinary approach in structuring the papers found. Perspectives from various fields were incorporated such as information technology, population health, and data science to ensure that the analysis was comprehensive and relevant to the topic. This approach allowed us to comprehensively identify and critically evaluate the decision context and capabilities of the systems. Furthermore, the multidisciplinary approach allowed us to identify gaps and limitations in current research and to provide recommendations for future research.

## Conclusion

PHM represents a data-driven approach to address the challenges faced by health care systems in worldwide. For this approach to succeed, a coherent data strategy is essential. Understanding the decision contexts in which BI systems are applied and identifying the users and their needs is crucial. Once this is clarified, the necessary BI capabilities can be derived. It is important to consider how partnerships between private and public organizations should be designed to protect intellectual property while enabling rapid knowledge development. To build knowledge systematically in public private collaboration, the development of an IDDA framework is proposed.

## Supplementary Material

ooae122_Supplementary_Data

## Data Availability

The authors confirm that the data supporting the findings of this study are available within the article.
