# Supplementary material for: Business intelligence systems for population health management: a scoping review
Source: JAMIA Open. 2024 Nov 27;7(4):ooae122. doi: 10.1093/jamiaopen/ooae122 (PMC11602128; doi:10.1093/jamiaopen/ooae122)
Supplement: ooae122_Supplementary_Data [file ooae122_supplementary_data.zip › RoordaSearchString.docx]

### ***Search strings***

### **Pubmed** ("Information Systems"[Mesh] OR "Informatics"[Mesh:NoExp] OR "Data Science"[Mesh] OR "Learning Health System"[Mesh] OR "information system*"[tw] OR "informatics"[tw] OR "Data Science*"[tw] OR "Learning Health System*"[tw] OR "Learning Healthcare System*"[tw] OR "Learning Health care System*"[tw] OR "PHI"[tiab] OR "PHIS"[tiab] OR "Data Management"[Mesh] OR "business intelligence"[tw] OR "data intelligence"[tw]) AND (population health[majr] OR "Population Health Management"[Majr] OR "population health"[ti] OR "PHM"[ti] OR "Accountable Care Organizations"[Majr]) NOT ("Clinical Studies as Topic"[Mesh] OR "Clinical Study" [Publication Type] OR "clinical study"[ti] OR trial[ti] OR RCT[ti])

### **EMBase** (exp "Information System"/ OR "Information Science"/ OR exp "Data Science" OR exp "Learning Health System"/ OR "information system*".ti,ab. OR "informatics".ti,ab. OR "Data Science*".ti,ab. OR "Learning Health System*".ti,ab. OR "Learning Healthcare System*".ti,ab. OR "Learning Health care System*".ti,ab. OR "PHI".ti,ab. OR "PHIS".ti,ab. OR exp "Information Processing"/ OR "business intelligence".ti,ab. OR "data intelligence".ti,ab.) AND (exp *"population health"/ OR exp *"Population Health Management"/ OR "population health".ti. OR "PHM".ti. OR exp *"Accountable Care Organizations"/) NOT ("Clinical Studies as Topic"/ OR "Clinical Study".pt. OR "clinical study".ti. OR trial.ti. OR RCT.ti.)

### **Web of science** TS=("information system*" OR "informatics" OR "Data Science*" OR "Learning Health System*" OR "Learning Healthcare System*" OR "Learning Health care System*" OR "PHI" OR "PHIS" OR "Data Management" OR "business intelligence" OR "data intelligence") AND TI=(population health OR "Population Health Management" OR "population health" OR "PHM" OR "Accountable Care Organizations") NOT TI=("Clinical Studies as Topic" OR "Clinical Study" OR "clinical study" OR trial OR RCT)
