# Supplementary figures and images for: Business intelligence systems for population health management: a scoping review
Source: JAMIA Open. 2024 Nov 27;7(4):ooae122. doi: 10.1093/jamiaopen/ooae122 (PMC11602128; doi:10.1093/jamiaopen/ooae122)

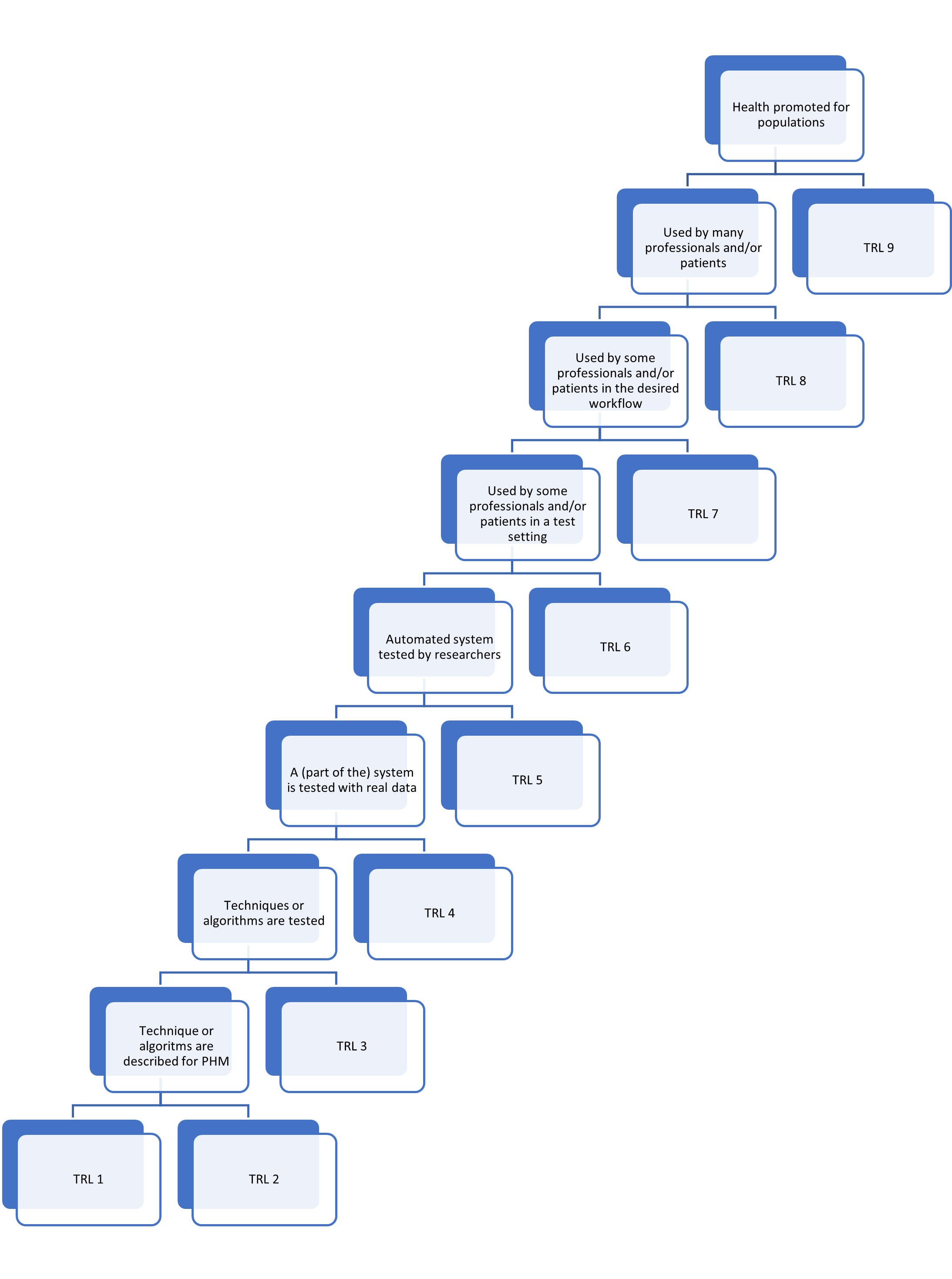

Supplement: ooae122_Supplementary_Data [file ooae122_supplementary_data.zip › Appendix_TRL_decision_tree.tif]
